# Molecular targeted agents as second-line treatment for hepatocellular carcinoma: a meta-analysis and review

**DOI:** 10.18632/oncotarget.21454

**Published:** 2017-10-03

**Authors:** Jung Han Kim, Bum Jun Kim, Hyun Joo Jang, Jin Lee

**Affiliations:** ^1^ Division of Hemato-Oncology, Department of Internal Medicine, Kangnam Sacred-Heart Hospital, Hallym University Medical Center, Hallym University College of Medicine, Seoul 07441, Republic of Korea; ^2^ Department of Internal Medicine, National Army Capital Hospital, The Armed Forces Medical Command, Sungnam 13574, Republic of Korea; ^3^ Division of Gastroenterology, Department of Internal Medicine, Dongtan Sacred-Heart Hospital, Hallym University Medical Center, Hallym University College of Medicine, Hwasung 18450, Republic of Korea

**Keywords:** hepatocellular carcinoma, targeted agent, second-line treatment, meta-analysis

## Abstract

It is unclear whether targeted agents can produce survival advantage in patients with advanced HCC previously treated with sorafenib. We performed this meta-analysis of randomized trials and reviewed clinical outcomes of molecular targeted agents in the second-line treatment for advanced HCC. A systematic computerized search of the electronic databases PubMed, Embase, Google Scholar, and Cochrane Library (up to May 2017) was carried out. From six studies, 2,388 patients were included in the meta-analysis. Almost all patients were treated with sorafenib as first-line therapy. Compared with placebo, targeted agents significantly improved time-to-progression (hazard ratio = 0.62, 95% confidence interval: 0.49–0.78, *P* < 0.0001). In terms of overall survival, targeted therapy tended to improve prognosis (hazard ratio = 0.86, 95% confidence interval: 0.74–1.01, *P* = 0.06). In conclusion, this meta-analysis indicates that molecular targeted agents have a potential to improve prognosis after failure of first-line treatment with sorafenib in patients with advanced HCC.

## INTRODUCTION

Hepatocellular carcinoma (HCC) is the fifth most common cancer worldwide [1–3]. Despite the recent advances in diagnostic and therapeutic modalities, HCC is still one of the major causes of cancer-related death [2, 3]. Surgical resection or ablation is the first choice of treatment for early stage HCC and chemoembolization is considered for patients with disease confined to the liver. However, locoregional therapies is possible in less than half of patients because of impairment of liver function caused by underlying cirrhosis or advanced disease at the time of diagnosis [4]. Moreover, about 70% of patients who underwent successful resection eventually relapse or develop de novo tumors [5].

For patients with advanced HCC, first-line treatment with sorafenib is recommended. Sorafenib is an oral, multi-targeted receptor tyrosine kinase inhibitors (TKIs) targeting vascular endothelial growth factor receptor (VEGFR), platelet-derived growth factor receptor (PDGFR), and RAF. In the SHARP trial, sorafenib showed a significant increase in overall survival (OS) (from 7.9 to 10.7 months, *P* < 0.001) and time-to-progression (TTP) (from 2.8 to 5.5 months, *P* < 0.001) compared with placebo [6]. Since these results with sorafenib were published in 2008, there have been no targeted agents to improve survival outcomes over sorafenib in the first-line treatment setting [7, 8]. Although sorafenib has significantly improved survival in advanced HCC, most patients show disease progression on/after or are intolerant to sorafenib in clinical practice. Therefore, there is an unmet need for effective salvage treatment after failure of sorafenib.

With more understanding of molecular mechanisms of pathogenesis, several novel targeted agents have been investigated in advanced HCC [8, 9]. Recently, a phase III placebo-controlled RESORCE trial reported that regorafenib significantly improved OS of patients with sorafenib-refractory HCC [10]. However, there has been a debate as to whether targeted agents can produce survival advantage in patients with advanced HCC previously treated with sorafenib. We performed this meta-analysis of randomized trials and reviewed clinical outcomes of molecular targeted agents as a second-line treatment for advanced HCC.

## RESULTS

### Results of search

**Figure 1:**
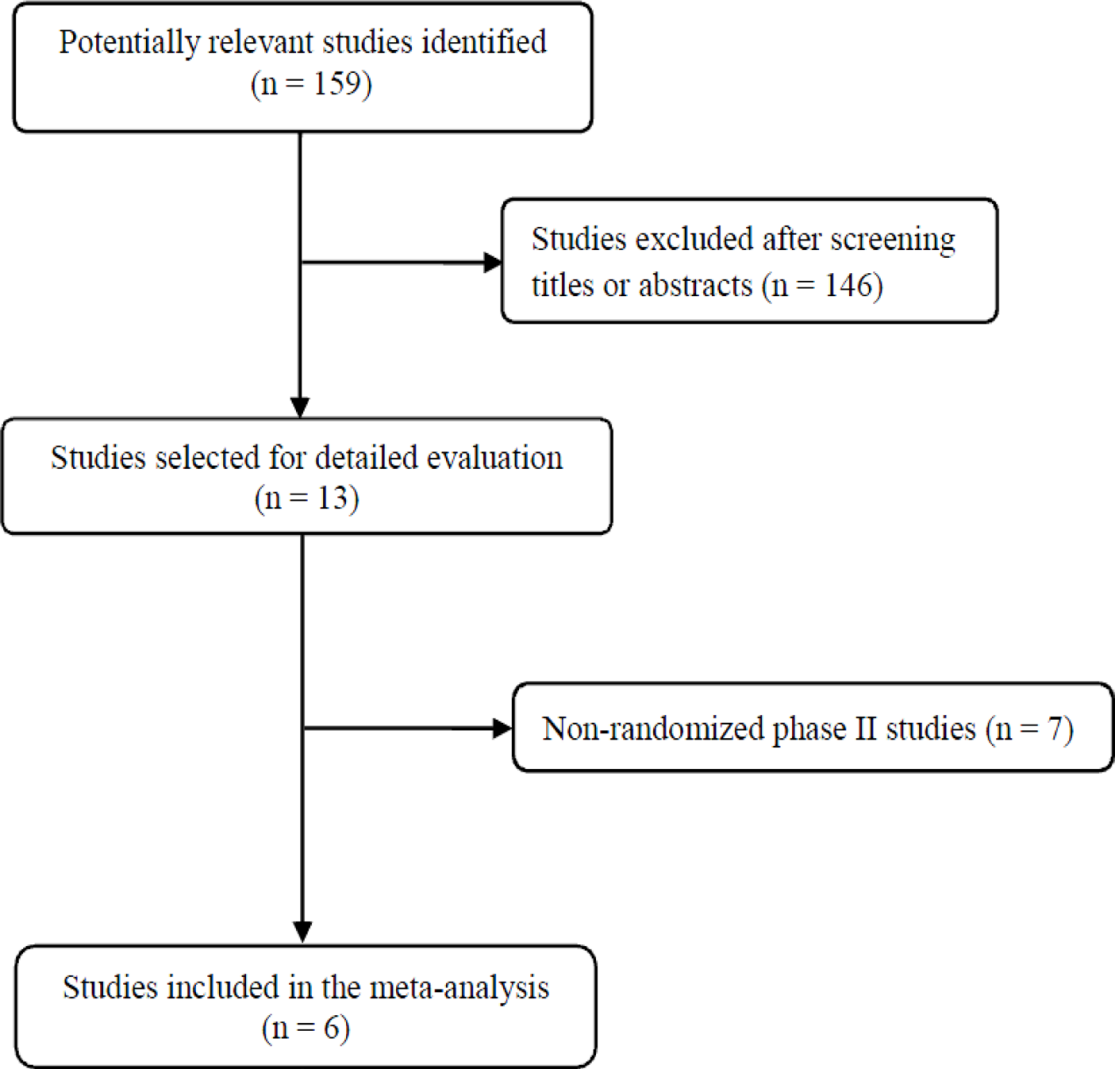
Flow diagram of search process

Figure 1 shows the flowchart of our study. A total of 159 potentially relevant studies were initially found, but 146 of them were excluded after screening the titles and abstracts. Of the remaining 13 potentially eligible studies, 7 were further excluded because they were non-randomized phase II trials. Finally, six studies were included in the meta-analysis [10–15].

### Characteristics of the included studies

Table 1 summarizes the main characteristics and clinical outcomes of the six included studies. There were four phase III trials [10, 11, 13, 15] and two phase II trials [12, 14]. From the six studies, 2,388 patients were included in the meta-analysis. Almost all patients (99%) were treated with sorafenib as first-line therapy.

**Table 1:** Summary of the six randomized studies comparing a targeted agent and placebo in second-line treatment setting for advanced hepatocellular carcinoma

First author (yr) Study	Phase	First-line Treatment	Treatment	Primary endpoint	No. of patients	ORR	Incidence of ≥ Gr 3 AEs	Median TTP (mo)	HR for TTP (95% CI)	Median OS (mo)	HR for OS (95% CI)
Llovet (2013) BRISK-PS	III	Sorafenib	Brivanib Placebo	OS	263 132	10% 2%	68% 38%	4.2 2.7	0.56 (0.42–0.76) *P* < 0.001	9.4 8.2	0.89 (0.69–1.15) *P* = 0.3307
Santoro (2013) APR 197-215	II	Sorafenib (103) Sunitibib (4)	Tivantinib Placebo	TTP	71 36	1% 0%	59% 9%	1.6 1.4	0.64 (0.43–0.94) *P* = 0.04	6.6 6.2	0.90 (0.57–1.40) *P* = 0.63
Zhu (2014) EVOLVE-1	III	Sorafenib	Everolimus Placebo	OS	362 184	2.2% 1.6%	71% 52%	3.0 2.6	0.93 (0.75–1.15) *P* = 0.01	7.6 7.3	1.05 (0.86–1.27) *P* = 0.68
Kang (2015)	II	Sorafenib (182)	Axitinib Placebo	OS	134 68	NA	82% 38%	3.6 1.9	0.62 (0.44–0.87) *P* = 0.004	12.7 9.7	0.91 (0.65–1.27) *P* = 0.287
Zhu (2015) REACH	III	Sorafenib	Ramucirumab Placebo	OS	283 282	6.7% 0.7%	41% 32%	2.8 2.1	0.63 (0.52–0.75) *P* < 0.0001	9.2 7.6	0.87 (0.72–1.05) *P* = 0.14
Bruix (2017) RESORCE	III	Sorafenib	Regorafenib Placebo	OS	379 194	10% 4%	67% 39%	3.2 1.5	0.44 (0.36–0.55) *P* < 0.0001	10.6 7.8	0.63 (0.50–0.79) *P* < 0.0001

TTP, time-to-progression; OS, overall survival; ORR, overall response rate; Gr, grade; AEs, adverse events; HR, hazard ratio; CI, confidence interval; NA, not available.

### Survival analyses

**Figure 2:**
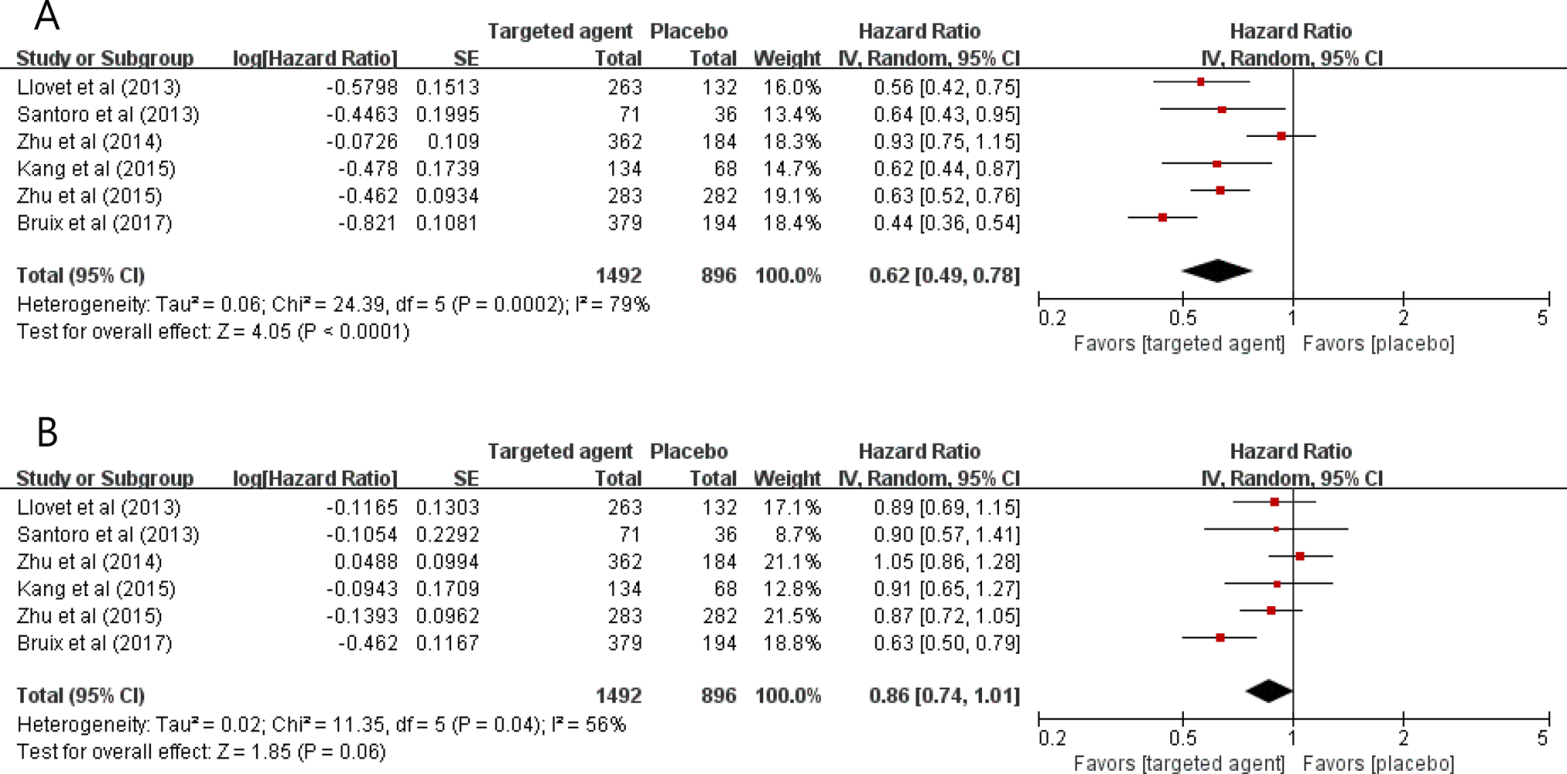
Forest plots for time-to-progression (A) and overall survival (B)

Compared with placebo, targeted agents significantly improved TTP [hazard ratio (HR) = 0.62, 95% confidence interval (CI): 0.49-0.78, *P* < 0.0001] (Figure 2A). We adopted the random-effects model because there was a significant heterogeneity (*X*^2^ = 24.39, *P* = 0.0002, *I^2^* = 79%).

In terms of OS, targeted therapy tended to prolong survival time (HR = 0.86, 95% CI: 0.74-1.01, *P* = 0.06), compared with placebo (Figure 2B). The random-effects model was used because there was a significant heterogeneity (*X*^2^ = 11.35, *P* = 0.04, *I^2^* = 56%).

### Publication bias

**Figure 3:**
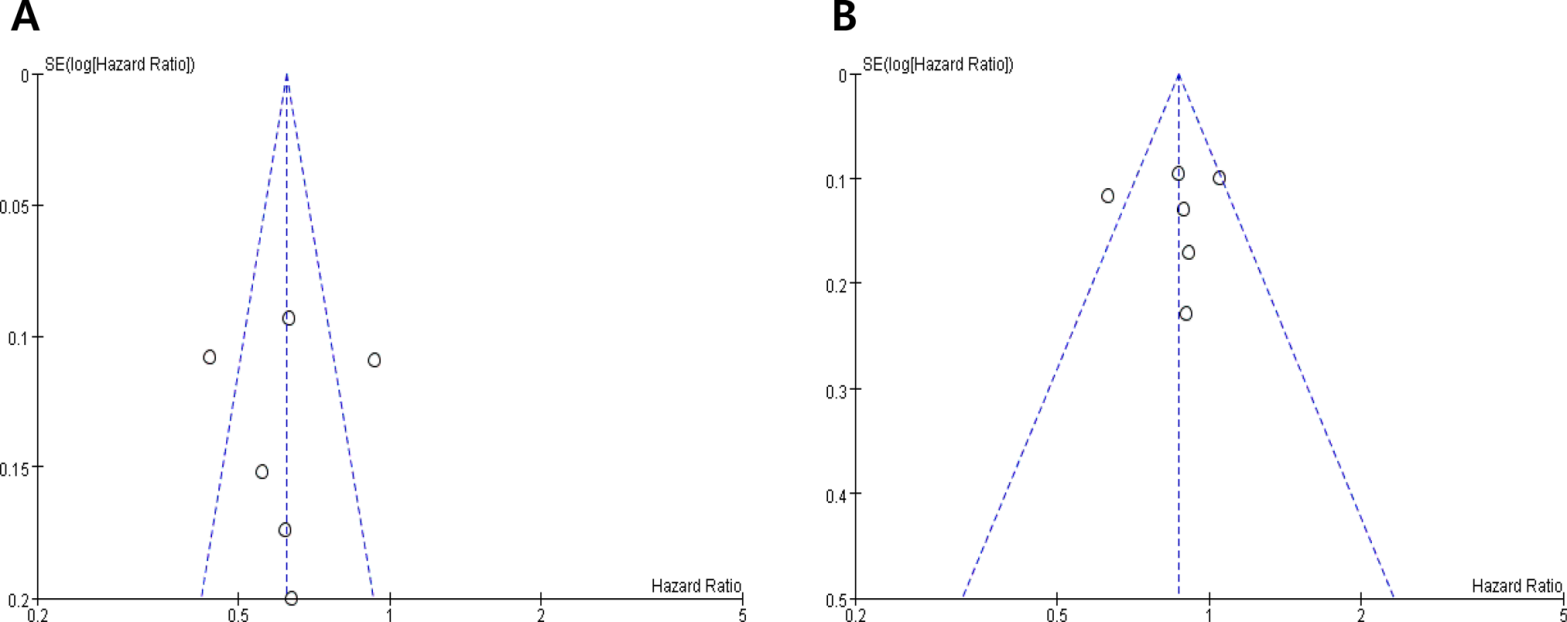
Funnel plots for publication bias regarding time-to-progression (A) and overall survival (B)

Begg's funnel plots and Egger's test showed no significant evidence of substantial publication biases for TTP (Begg's *P* = 0.174, Egger's *P* = 0.303) and OS (Begg's *P* = 0.425, Egger's *P* = 0.402) (Figure 3A and 3B).

## DISCUSSION

We performed this study to investigate the survival advantage of targeted agents as a rescue therapy after failure of first-line treatment in patients with advanced HCC. The meta-analysis of six randomized studies indicates that patients with advanced HCC that has progressed on sorafenib may have survival benefit from targeted agents.

Patients with advanced HCC usually show a poor prognosis with few systemic therapeutic options. A considerable number of molecular targeted agents with different mechanisms of action have been tested in advanced HCC: however, most of them have shown a limited clinical value. As of now, sorafenib is the only agent approved by the U. S. Food and Drug Administration (FDA) for the first-line therapy of patients with advanced HCC. However, the efficacy of sorafenib is usually short-lived and considerable number of patients experience severe toxicities [6, 16]. Outcomes in the setting of sorafenib failure (resistance or intolerance) are poor, with a median OS for placebo arms of second-line trials in the range of 7 to 8 months. Until recently, however, no established agents have been existed for the treatment of patients with advanced HCC after failure of sorafenib.

There are four randomized trials that have tested a targeted agent as rescue therapy in comparison with placebo in patients with sorafenib-refractory HCC [10, 11, 13, 15]. Within the past few years, three candidate agents including brivanib (BRISK-PS), everolimus (EVOLVE-1), and ramucirumab (REACH) failed to meet the primary endpoint (OS) in placebo-controlled, phase III trials [11, 13, 15]. Brivanib is a dual inhibitor of the VEGFR- and FGFR-mediated pathways. In a phase II trial, brivanib showed a promising activity with a median OS of 9.8 months in patients with HCC resistant to anti-angiogenic agents [17]. In the subsequent phase III BRISK-PS study of 395 patients with advanced HCC who were resistant or intolerant to sorafenib, however, the drug showed no significant survival benefit versus placebo [11]. Compared with placebo, brivanib significantly improved overall response rate (ORR) (10% vs. 2%, *P* = 0.003) and TTP (median 4.2 vs. 2.7 months, HR = 0.56, 95% CI: 0.42-0.76, *P* < 0.001). However, the drug failed to meet the primary endpoint of OS improvement (median 9.4 vs. 8.2 months, HR = 0.89, *P* = 0.3307). The EVOLVE-1 study evaluated the mTOR inhibitor everolimus versus placebo in patients with advanced HCC who failed sorafenib [13]. Despite antitumor activity in phase I/II studies, there was no significant difference in OS between everolimus (median 7.6 months) and placebo (median 7.6 months) (HR = 1.05, 95% CI: 0.86–1.27, *P* = 0.68). Ramucirumab is a recombinant monoclonal antibody to VEGFR-2. As an inhibitor of angiogenesis, ramucirumab was expected to be effective in HCC, which is a highly angiogenic tumor. In the phase III REACH trial, ramucirumab was associated with a significant improvement in PFS (median 2.8 vs. 2.1 months, HR = 0.63, *P* < 0.001) [15]. However, the drug also failed to observe a significant OS benefit versus placebo (median 9.2 vs. 7.6 months, HR = 0.87, 95% CI: 0.72–1.05, *P* = 0.14).

There are two randomized phase II trials of novel targeted agents in the second-line treatment setting of advanced HCC [12, 14]. Tivantinib is a selective inhibitor of c-Met. c-Met is the product of the proto-oncogene *MET* and the tyrosine kinase receptor for hepatocyte growth factor (HGF). The c-Met/HGF signaling pathway is implied in carcinogenesis and progression of HCC [18, 19].In a randomized phase II trial in patients with advanced HCC for whom sorafenib or sunitinib failed, tivantinib showed a significant improvement in TTP (primary endpoint) compared with placebo (median 1.6 vs. 1.4 months, HR = 0.64, 95% CI: 0.43–0.94, *P* = 0.04) [12]. Axitinib is a selective inhibitor of VEGFRs 1-3. In a randomized phase II trial by Kang *et al.*, axitinib as second-line therapy for advanced HCC significantly improved TTP compared with placebo (median 3.6 vs. 1.9 months, HR = 0.62, 95% CI: 0.44–0.87, *P* = 0.004) [14]. However, the drug failed to achieve the primary endpoint of OS improvement (median 12.7 vs. 9.7 months, HR = 0.91, *P* = 0.287).

Several plausible hypotheses may be proposed to explain reasons for these failures in the second-line treatment for advanced HCC. First, the failures might be related to the high molecular heterogeneity of HCC. Second, selection bias between treatment arms caused by inadequate patient stratification might be attributable to the negative results. Third, insufficient exploration of liver toxicities in phase II trials may have led to more discontinuations of test drugs than initially anticipated. Finally, poor performance status of patients may inevitably affect the survival data.

On April 27, 2017 the FDA approved the use of regorafenib for patients with advanced HCC who have been previously treated with sorafenib. Regorafenib is an oral multi-kinase inhibitor that blocks VEGFR, PDGFR, RET, c-KIT, BRAF, and fibroblast growth factor receptor (FGFR). The approval was based on the RESORCE study of 573 patients with documented disease progression following sorafenib [10]. Patients were randomly allocated to receive regorafenib 160 mg orally once daily plus best supportive care (BSC) or matching placebo with BSC for the first 21 days of each 28-day cycle. The drug significantly increased OS (median 10.6 vs. 7.8 months, HR = 0.63, 95% CI: 0.50–0.79, *P* < 0.0001) and progression-free survival (PFS) (median 3.1 vs. 1.5 months, HR = 0.46, 95% CI: 0.37–0.56, *P* < 0.0001) compared with placebo. The common adverse events observed in 20% or more of patients included pain, hand-foot skin reaction, fatigue, diarrhea, decreased appetite, hypertension, infection, dysphonia, elevated bilirubin, fever, mucositis, weight loss, rash, and nausea.

In the current meta-analysis of the six randomized, placebo-controlled trials in HCC patients who failed first-line therapy (mostly sofrafenib), molecular targeted agents significantly improved TTP (HR = 0.62, *P* < 0.0001). In terms of OS, targeted therapy tended to improve prognosis (HR = 0.86, *P* = 0.06), compared with placebo. These findings suggest that targeted agents may be a considerable therapeutic option for patients with advanced HCC that has progressed on sorafenib. Because four individual studies failed to meet the primary endpoint of OS improvement [11–15], however, there seem to be a critical need to identify biomarkers that can predict the efficacy of each targeted agent.

To date, no definite biomarkers are available to predict the benefit of molecular targeted agents in the treatment of HCC. Because of molecular heterogeneity of HCC, the identification of key oncogenic pathways that would guide selection of targeted therapy may be challenging. However, there have been some encouraging findings to identify candidates who would most likely benefit from targeted agents. In the subgroup analysis of the phase III REACH trial, patients with elevated baseline levels of serum alpha-fetoprotein (AFP ≥ 400 ng/mL), an adverse prognostic maker, benefited from ramucirumab (median OS 7.8 vs. 4.2 months, HR = 0.67, 95% CI: 0.51–0.90, *P* = 0.0059) [15]. High AFP expression may be associated with increased angiogenesis and possibly enhanced sensitivity to VEGFR-2 inhibition. In the phase II trial of tivantinib, patients with c-Met-high tumor showed longer TTP (median 2.7 vs. 1.4 months, HR = 0.43, 95% CI: 0.19–0.97, *P* = 0.03) and OS (median 7.2 vs 3.8 months, HR = 0.38, 95% CI: 0.18-0.81, *P* = 0.01) than those in the placebo group [12]. However, patients with low-c-Met tumor showed no advantage from tivantinib (median TTP, 1.5 vs. 1.4 months, HR = 0.96, *P* = 0.92; median OS, 5.0 vs. 9.0 months, HR = 1.33, *P* = 0.92).

Our meta-analysis has several inherent limitations that need to be noted. First, the small number of included studies is a major limitation of this meta-analysis. Second, the individual studies had been conducted with various targeted agents with different mechanisms of action. Third, the impact of targeted therapy on quality of life could not be analyzed due to the lack of available data. Finally, papers published only in English were included, which might bias the results.

In conclusion, this meta-analysis indicates that targeted agents have a potential to improve prognosis in patients with advanced HCC after failure of first-line treatment with sorafenib. These results suggest that subsequent targeted therapy may be a considerable option for HCC patients who progressed during or after sorafenib treatment or were intolerant to the drug. However, there is an urgent need to discover biomarkers that can predict the efficacy of each targeted agent.

## MATERIALS AND METHODS

### Publication searching strategy

We performed this study according to the Preferred Reporting Items for Systematic Reviews and Meta-Analyses (PRISMA) guidelines [20]. A systematic computerized search of the electronic databases PubMed, Embase, Google Scholar, and Cochrane Library (up to May 2017) was carried out. We also reviewed abstracts presented in the American Society for Clinical Oncology (ASCO) Annual Meeting, ASCO Gastrointestinal Cancers Symposium, and European Society for Medical Oncology (ESMO) Congress. The search used the following keywords: “hepatocellular carcinoma or hepatoma or liver neoplasm,” and “second-line,” and “randomized.” The related articles function in PubMed was also used to identify all relevant articles.

### Inclusion criteria

Eligible studies should meet the following inclusion criteria: (i) randomized, controlled phase II or III trials; (ii) patients with previously treated advanced HCC; (iii) randomization of patients to either a targeted agent or best supportive care with or without placebo; (iv) HRs and their 95% CIs for TTP and/or OS provided.

### Data extraction

Data extraction was carried out independently by two investigators (BJK and HJJ). If these two authors did not agree, the principle investigator (JHK) was consulted to resolve the dispute.

The following data were extracted from all eligible studies: first author's name; year of publication; study name; trial phase; number of patients; treatments; overall response rate; severe adverse events; median TTP and OS, including HRs and 95% CIs.

### Statistical analysis

The statistical values were obtained directly from the original articles. The effect size of TTP and OS was pooled through HR and its 95% CI. The RevMan software (version 5.2) was used to combine the data. Heterogeneity across studies was examined by the Q statistic and the I^2^ inconsistency test. The fixed-effects model (Mantel–Haenszel method) was selected for pooling homogeneous outcomes when *P* ≥ 0.1 and I^2^ ≤ 50%, and the random-effects model (DerSimonian–Laird method) was used for pooling heterogeneous outcomes when *P* < 0.1 and I^2^ > 50%.

The plots show a summary estimate of the results from all the studies combined. The size of each square represents the estimate from each study and reflects its statistical ‘weight.’ Results are presented as forest plots, with diamonds representing estimates of the pooled effect and the width of each diamond representing its precision. The line of no effect is number one for binary outcomes, which depicts statistical significance if not crossed by the diamond [21]. All reported *P*-values were two-sided and *P* < 0.05 was considered statistically significant.

The possibility of publication bias was assessed with visual inspection of the funnel plots [22] and by performing Egger's test [23].
